# Reduced Pathogen‐Specific T Cell Response With BCMA‐Targeted T Cell‐Engaging Therapy to Multiple Myeloma

**DOI:** 10.1002/jha2.70388

**Published:** 2026-09-21

**Authors:** Ida Steiro, Synne Stokke Tryggestad, Eli Svorkdal Hess, Hanne Hella, Pegah Abdollahi, Kari Lenita Falck Moore, Margrete Friestad, Magne Børset, Tobias S. Slørdahl, Anne Marit Sponaas

**Affiliations:** ^1^ Department of Clinical and Molecular Medicine Faculty of Medicine and Health Sciences Norwegian University of Science and Technology (NTNU) Trondheim Norway; ^2^ Oslo Myeloma Center Department of Hematology Oslo University Hospital Oslo Norway; ^3^ KG Jebsen Center for B cell Malignancies Institute of Clinical Medicine University of Oslo Oslo Norway; ^4^ Department of Hematology and Oncology Stavanger University Hospital Stavanger Norway; ^5^ Institute of Clinical Medicine University of Oslo Oslo Norway; ^6^ Department of Immunology and Transfusion Medicine St. Olav's Hospital – Trondheim University Hospital Trondheim Norway; ^7^ Department of Hematology St. Olav's Hospital – Trondheim University Hospital Trondheim Norway

## Abstract

**Introduction:**

Bispecific antibody (BsAb) treatment can lead to increased infections in myeloma patients.

**Methods:**

We used ELISpot and flow cytometry to test the T cell response to BCMA‐CD3‐bispecific T cell engager (BiTE) pavurutamab (PAV) and BCMA‐expressing myeloma cells after in vitro culture.

**Results:**

Reduced pathogen‐specific responses were found in T cells cultured with PAV yet they killed myeloma cells. In an exploratory study, reduced pathogen‐specific memory T cell responses were detected in PBMCs from four myeloma patients responding to BCMA‐targeted BsAb therapy.

**Conclusion:**

Our preliminary observation and its link to infection should be followed up in a larger study.

**Trial Registration:**

The authors have confirmed clinical trial registration is not needed for this submission.

## 1 Introduction

Bispecific antibodies (BsAb) are effective in treatment of relapsed and refractory multiple myeloma, but can lead to adverse effects including increased rates and severity of infections, particularly where BCMA is targeted [1]. This has been attributed to BCMA expression on B cells, but could also be due to loss of T cell fitness [2]. Little is known about how BsAb treatment affects circulating memory responses. We tested antigen‐specific responses to common pathogens in T cells cocultured with BCMA‐expressing human myeloma cells lines (HMCLs) and pavurutamab (PAV), an anti‐CD3‐BCMA bispecific T cell engager (BiTE) [3]. To avoid confounding effects of differential T cell fitness in patient samples, we used T cells from healthy donors. We investigated how well the T cells were able to respond to common pathogen antigens in an ELISpot assay and their ability to kill myeloma cells in vitro.

## 2 Materials and Methods

*IFN‐γ T cell ELISpot assay*: T cells isolated from the PBMC‐HMCL cocultures were seeded in R4 medium with or without APCs (antigen presenting cells) in a T cell:APC ratio of 1:0.5 with 1 µg/mL CEFX peptide pool and 20 U/mL IL‐2. After 2 days IFN‐γ‐producing T cells were quantified by ELISpot assay, using manufacturer's protocol; 5 × 10^4^ cells/well (T cells and APCs or T cells only) were seeded in 96‐well ELISpot plates, precoated with anti‐IFN‐γ antibody in R2 medium. Cells were stimulated with CEFX peptide pool 0.75 µg/mL, 0.3 µg/mL anti‐CD3 mAb or media. Plates were incubated at 37°C with 5% CO_2_ for 21–24 h followed by ALP‐linked immunosorbent detection. The AID vSpot Spectrum was used for counting spots. Results are expressed as number of spot‐forming colonies (SFCs)/5 × 10^4^ seeded cells. PBMCs from a healthy donor were added with each experiment as positive assay control (Figure S1A,B). Details of methods are provided in Supporting Information.

## 3 Results

### 3.1 PAV Stimulation Reduced Pathogen‐Specific T Cell Responses in Vitro But the T Cells Killed Myeloma Cells

**FIGURE 1:**
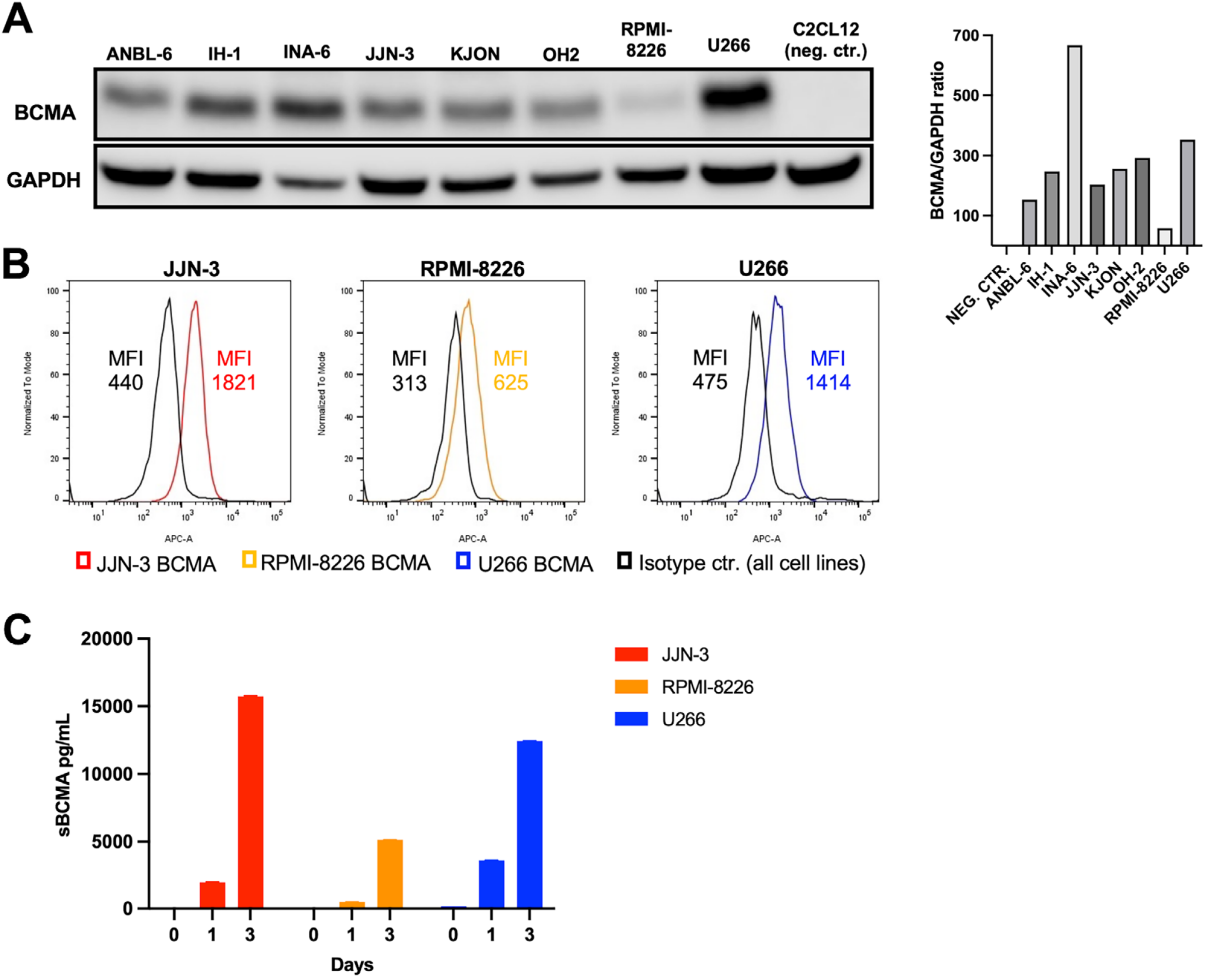
BCMA expression and sBCMA. (A) BCMA protein expression was investigated in eight myeloma cell lines by Western blotting. Bar graphs are plotted relative to the myoblast cell line C2CL12 which was used as negative control, and which value is set to 1. The IL‐6 independent cell lines JJN‐3, RPMI‐8226, and U266 was used for further experiments. (B) BCMA surface expression was investigated by flow cytometry. (C) sBCMA from JJN‐3, RPMI‐8226, and U266 was determined by ELISA. 2 × 10^5^ HMCLs were cultured and supernatants harvested at baseline (Day 0), and after 1 and 3 days. One out of at least two independent experiments is shown in (A), (B), and (C). HMCL: Human myeloma cell line, MFI: Median fluorescence intensity, sBCMA: soluble BCMA.

Healthy individuals have circulating memory T cells to pathogens they have been exposed to or vaccinated against [4]. Pathogen‐specific, IFN‐γ‐producing CD8+ T cells can be detected in PBMCs with ELISpot assays after stimulation with nonameric peptides from many pathogens (Figure S1A,B) [5]. To determine whether BiTE stimulation influences antigen‐specific T cell responses, PBMCs were incubated with PAV or BiTE isotype control antibody (control) and HMCLs as described in Methods. Three BCMA‐expressing HMCLs were used; JJN‐3, RPMI‐8226, and U226 (Figure 1A–C).

**FIGURE 2:**
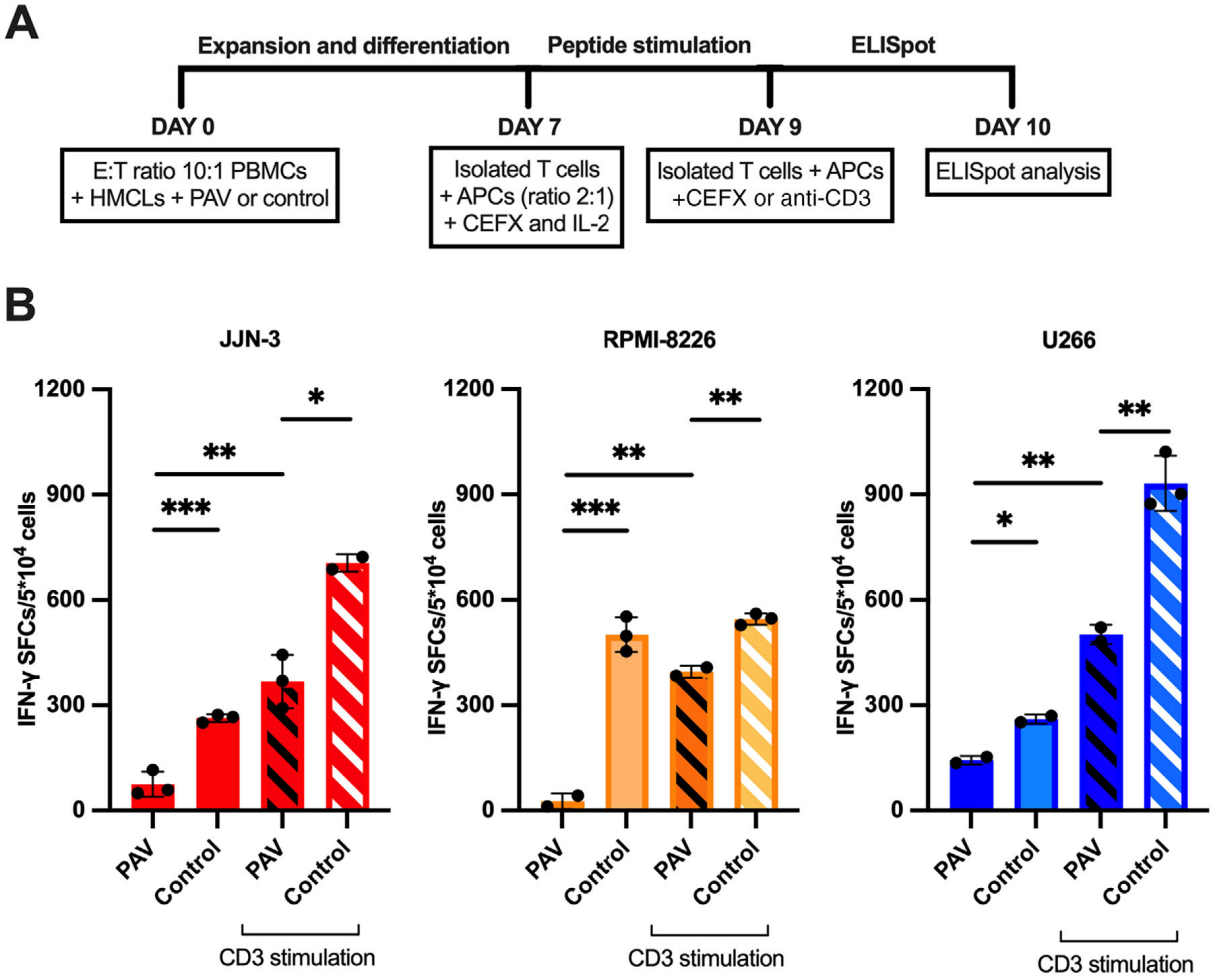
Pavurutamab stimulation suppresses pathogen‐specific T cell activation. (A) Experimental setup for (B). PBMCs were seeded with HMCLs and pavurutamab (PAV) or BiTE isotype control (control) antibody on Day 0. After incubation for 7 days, T cells were isolated from both conditions and seeded with syngeneic APCs, CEFX peptide pool, and IL‐2. After another 2 days, the cells were harvested and restimulated with CEFX or CD3 antibody in ELISpot plates. Following overnight incubation, the plates were developed, and spots counted on AID vSpot Spectrum. (B) Number of SFCs with T cells isolated from coculture with JJN‐3, RPMI‐8226, and U266 myeloma cell lines. Bar graphs show IFN‐γ production from T cells treated with PAV or control antibodies and stimulated with CEFX (two left bars) or anti‐CD3 (two right bars). Data in (B) represent the mean + SD of triplicates of one representative out of at least three independent experiments. *p*‐values were calculated by unpaired Student's *t*‐test. SFC: Spot forming colony. ns = not significant (*p* ≥ 0.05), *p < 0.05, **p < 0.01, ***p < 0.001.

BsAb is given with weekly and forthnightly intervals. To model the response after a week, T cells were isolated from cultures after 7 days, seeded with syngeneic APCs and CEFX peptides before they were tested in an ELISpot assay (Figure 2A). Although more T cells were harvested from cultures stimulated with PAV than control (Figure S2), PAV significantly reduced the number of pathogen‐specific T cells (Figure 2B). The PAV‐stimulated T cells responded to anti‐CD3, albeit with fewer SFCs than those cultured with control (Figure 2B). A much lower level of response to both CEFX and anti‐CD3 was observed on Day 10 in the absence of APCs at Day 7 (Figure S3A,B), suggesting that restimulation with peptide on APCs was necessary to obtain adequate numbers of IFN‐γ‐producing CD8+ T cells. It was difficult to model the clinical response to repeated injections as in vitro stimulations with PAV beyond Day 7 also led to T cell death. On Day 7 after a second restimulation, there were only 7%–8% viable T cells (Figure S4). This made it very difficult to recover enough T cells to test functional responses.

**FIGURE 3:**
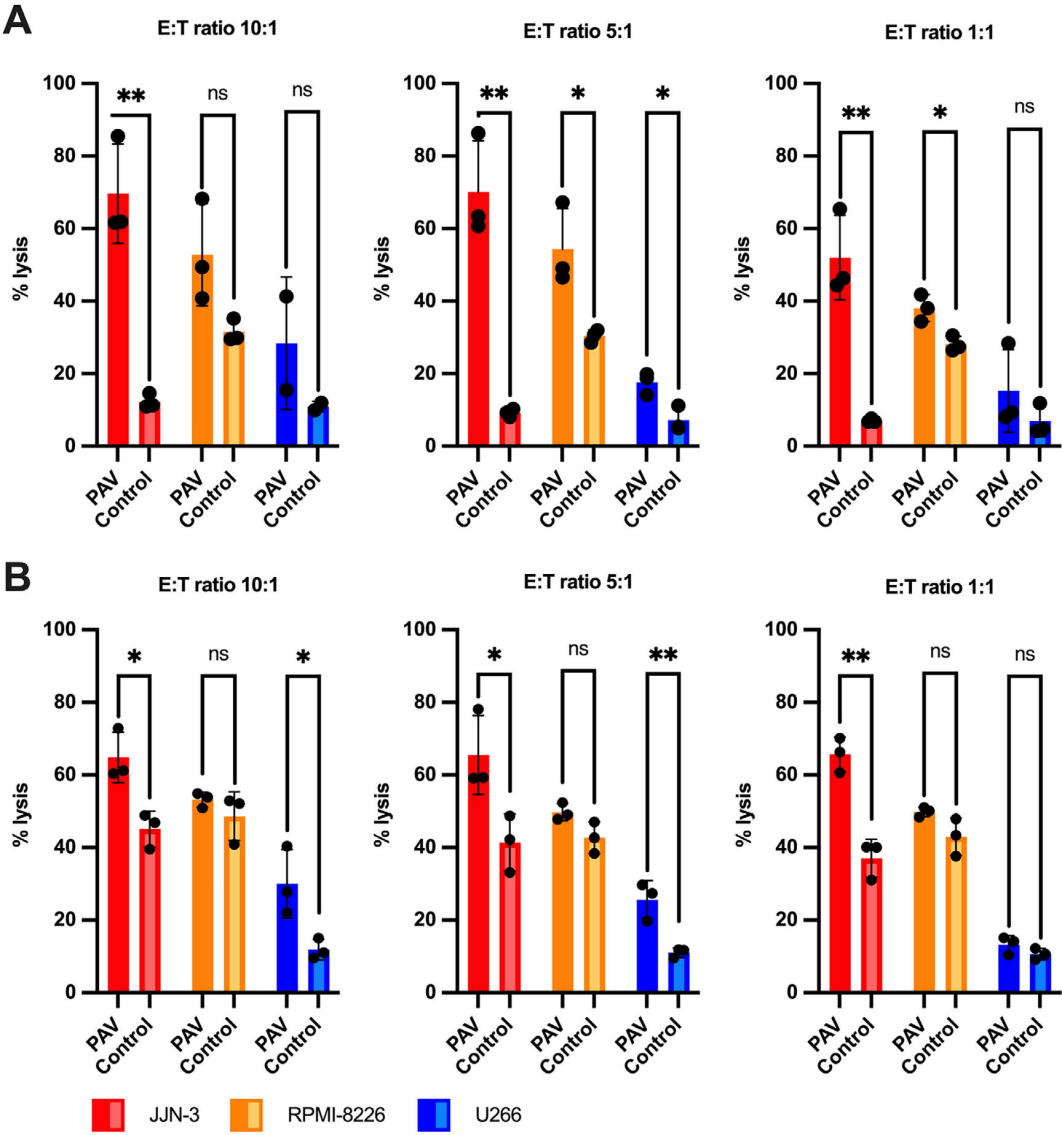
Cytotoxic efficacy in pavurutamab‐treated cells. Killing ability in T cells treated with pavurutamab (PAV) or BiTE isotype control (control) antibody and HMCLs at indicated target‐effector (E:T) ratios at baseline (A) and following restimulation (B). Percent lysis (dead myeloma cells) was calculated based on percentages obtained in the FlowJo software. The figure shows mean + SD of singlicates from at least two independent experiments performed with one representative PBMC donor. In total the experiment was performed with three different donors. *p*‐values were calculated by unpaired Student´s *t*‐test. ns = not significant (*p* ≥ 0.05), *p < 0.05, **p < 0.01. Results are also shown in Table S2 and gating strategy in Figure S5.

Exhausted or activation‐induced dysfunctional T cells have attenuated effector functions, including impaired killing [6]. We investigated the ability to kill myeloma cells before and after culture and restimulation with PAV (Figure 3A,B, Figure S5). All cell lines were lysed after 24 h incubation with PAV and T cells (Figure 3A), and T cells isolated from 7‐day cocultures with PAV killed HMCLs more efficiently than control when JJN‐3 and U266 cells were targeted (Figure 3B, Table S1). RPMI‐8226 were lysed by restimulated T cells regardless of exposure to PAV or control.

### 3.2 PAV Upregulated Activation Markers on T Cells

Reduction in the number of CEFX specific IFNγ spots (Figure 2B) could be caused by inadequate activation or activation‐induced dysfunction of T cells.

**FIGURE 4:**
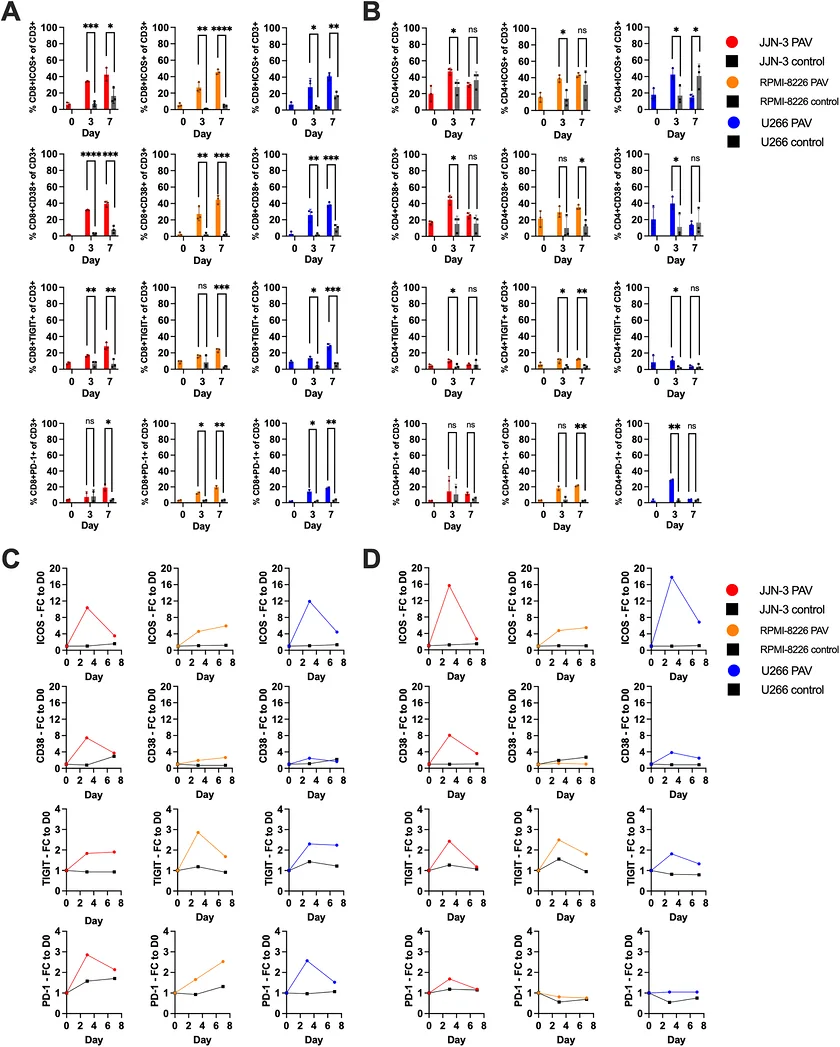
Pavurutamab upregulates activation and exhaustion markers on T cells. PBMCs from healthy donors were seeded with HMCLs and pavurutamab (PAV) or BiTE isotype control (control) antibody. The coculture was harvested and stained for analysis by flow cytometry at baseline (Day 0), and after 3 and 7 days cultivation, respectively. ICOS, CD38, TIGIT, and PD‐1 surface markers were investigated. (A) and (C) display CD8+ subpopulations of CD3+ cells, and (B) and (D) show CD4+ subpopulations of CD3+ cells. Figures show time course of expression as percentage positive cells (A, B) and as MFI fold change relative to baseline samples obtained from analysis in the FlowJo software (C, D). Figures (A, B) show mean ± SD of singlicates from three independent experiments performed with one representative out of three PBMC donors (C, D). In total, the experiment was performed with three different PBMC donors. Statistical significance was calculated by unpaired Student's *t*‐test (A, B). FC: Fold change. ns = not significant (*p* ≥ 0.05), *p < 0.05, **p < 0.01, ***p < 0.001, ****p < 0.0001. Gating strategy is shown in Figure S7.

Reduced T cell function with altered expression of surface markers were demonstrated for BsAbs [7]. We determined activation and exhaustion markers at baseline and after 3‐ and 7‐day culture with PAV or control and HMCLs. PBMCs cultured without HMCLs did not upregulate any of the markers (Figure S6). Compared with control, PAV increased expression (% positive cells and MFI) of ICOS, CD38, TIGIT, and PD‐1 (Figure 4, Figure S7). Higher proportions of CD8 T cells expressed these markers than CD4+ cells, possibly reflecting a dominant role of CD8+ cells upon BiTE stimulation [8]. When presenting the data as fold change MFI, for some of the cell lines and markers, there was a trend in which expression peaked at Day 3 of stimulation, followed by a decline at Day 7 (Figure 4C,D, Figure S7). TIGIT and PD‐1 peaked at Day 3 and either declined or was unchanged after 1‐week. Therefore, PAV activated the T cells and the suppression of the IFN‐γ response on Day 7 was not due to lack of stimulation.

Soluble BCMA (sBCMA) reduces the response to BCMA‐targeted BsAb‐therapy [9]. sBCMA was found in culture supernatants from all cell lines (Figure 1C) and in the cocultures (Figure S8A,B). However, very little sBCMA was detected in the cultures with PAV. Importantly, PAV expanded T cell numbers (Figure S2), and upregulated activation molecules on T cells, which was not seen in cultures with control. Therefore, sBCMA was unlikely to inhibit PAV and generation of CD8+ IFN‐γ producing T cells in these in vitro studies.

### 3.3 Myeloma Patients Receiving BCMA‐BsAb Have Reduced Numbers of Antigen‐Specific T Cells in Circulation

**FIGURE 5:**
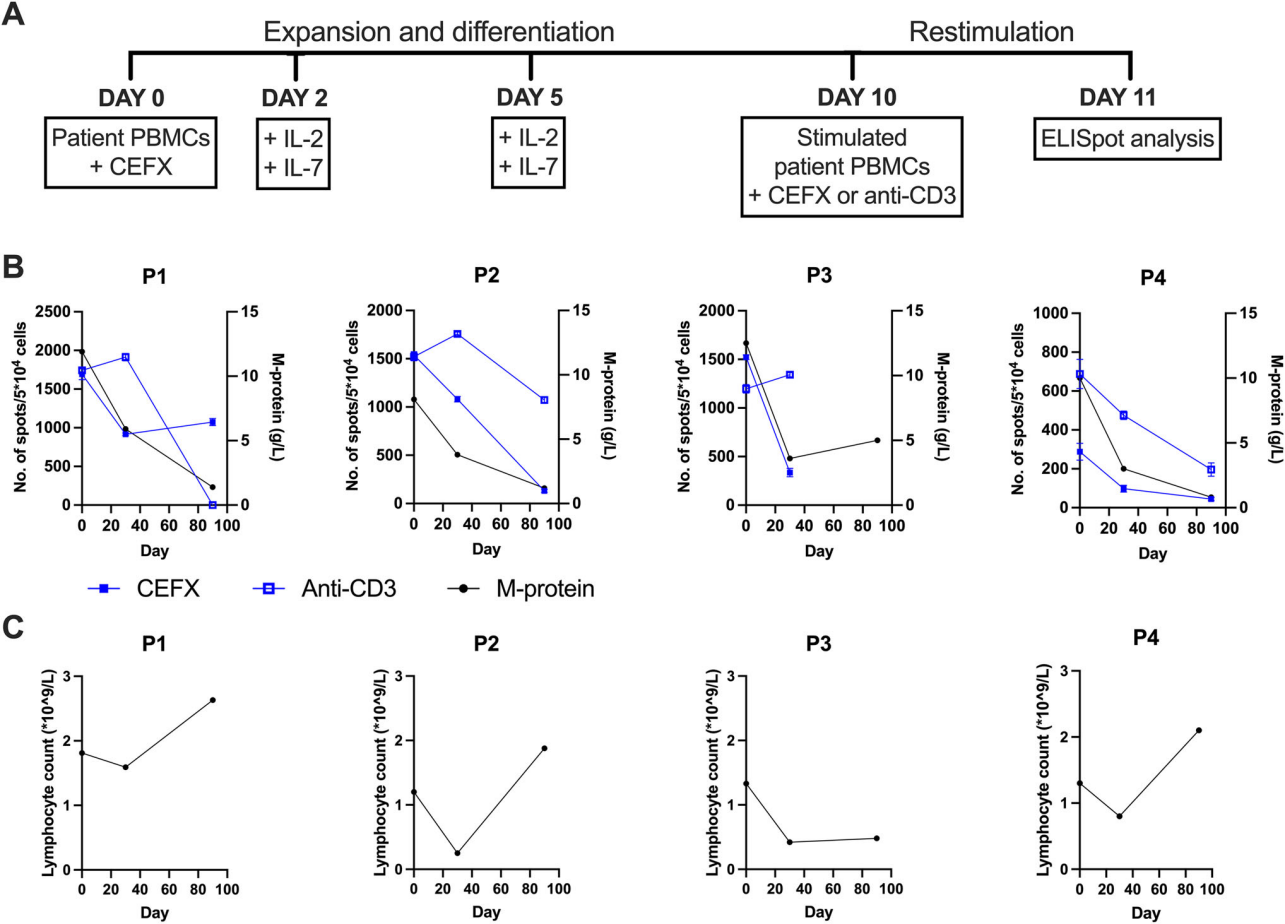
Memory T cell responses and M‐protein measurements in BCMA BsAb‐treated MM patients. (A) Experimental setup for (B). PBMCs from patients on BCMA‐targeted BsAb‐treatment (P1‐4) were isolated before treatment and at Days 30 and 90 and cultured with CEFX peptide mix from experiment Day 0. Recombinant IL‐2 and IL‐7 were added on Days 2 and 5. After 10 days, cells were harvested and seeded in ELISpot plates with CEFX or anti‐CD3. After 24 h incubation, the plate was developed and spots counted on AID vSpot Spectrum. (B) Graphs show merged data of pathogen‐specific memory CD8+ T cell responses to CEFX (blue) and anti‐CD3 (blue outline), and M‐protein values (black). CEFX and anti‐CD3 are read on the left *y*‐axis, and M‐protein is read on the right *y*‐axis. The graphs show mean + SD of three technical replicates (left *y*‐axis). In Donor 3 (P3), no PBMCs were available for analysis at Day 90. (C) Lymphocyte counts in patients at baseline (Day 0), and after 30 and 90 days on treatment. SFC: Spot forming colony.

Antigen‐specific memory T cell responses can be detected in human PBMCs with an ELIspot assay after in vitro culture with IL‐2, IL‐7, and antigen stimulation (Figure 5A) [ 5]. CEFX‐specific memory T cell responses were measured in PBMCs collected from four MM patients treated with BCMA‐directed BsAbs at the start, after 30 and 90 days (± 7 days). The number of pathogen‐specific IFN‐γ‐secreting CD8+ T cells dropped during treatment (Figure 5B). A drop at Day 30 (Figure 5B) could be explained by T cell margination during the first cycle [10] as suggested from the decrease in total lymphocyte count (Figure 5C), but the T cells still responded to anti‐CD3. Nevertheless, at Day 90 the lymphocyte number increased and the pathogen‐specific T cells remained low. Incidentally, all patients suffered from infections (Table 1). Although only exploratory, these data may suggest that pathogen‐specific memory T cell responses decline in patients treated with at least two cycles of BCMA‐directed therapy.

**TABLE 1:** Clinical data of MM patients.

General				
Patient ID	P1	P2	P3	P4
Age	72	49	47	68
Gender	M	M	M	F
Time since diagnosis	6,2	3,6	4,8	0.8
ISS at diagnosis	II	II	II	II
R‐ISS at diagnosis	N/A	II	II	N/A
Extramedullary disease at diagnosis	No	No	No	No
Most recent FISH analysis	ND*	ND*	del(17p)**	ND***
M‐protein	IgG‐kappa	IgG‐kappa	IgG‐lambda	IgG‐kappa
Previous LOT	2	2	2	0
ASCT	Yes	Yes	Yes	Yes
Proteasome inhibitor	1	1	2	1
Immunomodulatory drug	1	1	1	1
Anti‐CD38 antibody	0	0	0	0
Treatment start date	30/05/2023	25/09/2023	16/10/2023	07/10/2024
M‐protein at start of treatment†	12,5	8,3	11,9	10
Bone marrow at start of treatment (% PCs)	Aspirate 12%, biopsy 20%.	Aspirate 17%, biopsy NA.	Aspirate 2%, biopsy 5%.	Aspirate NA, biopsy 10%–15%
Best response	MRD‐negative sCR	sCR	MRD‐negative sCR	sCR
Still on treatment at cutoff	Yes	Yes	Yes	Yes
Days follow‐up	368	250	229	609
Hypogammaglobulinemia	Yes	Yes	Yes	Yes
No. of infections	2	4	2	10
Type of infection	RTI	RTI	Skin infection, RTI	RTI, UTI
Highest grade infection	II	II	II	III
Neutropenia	III	III	IV	IV
Leukopenia	III	III	III	III
Lymphopenia	IV	IV	IV	IV
IgG‐substitution	Yes	Yes	Yes	Yes
Pneumocystis	Yes	Yes	Yes	Yes
Herpes zoster	Yes	Yes	Yes	Yes
Pneumococcal vaccine within the last 6 years	No	Yes	Yes	Yes
Influenza vaccine the previous season	No	No	Yes	Yes
COVID‐19 vaccination (no. of doses)	3	4	5	5

*Note*: FISH abnormalities: *Tested for t(4;14), t(11;14), t(14;16), t(14;20), del(17p), 1q21+. **Tested for t(4;14), t(14;16), del(17p), 1q21+. ***Tested for del(17p), 1q21+, del(1p32.3), del(13q14), IGH rearrangement (14q32).

Abbreviations: ASCT, autologous stem cell transplantation; LOT, lines of treatment; MRD, minimal residual disease; PCs, plasma cells, RTI, respiratory tract infection; sCR, stringent complete response; UTI, urinary tract infection.

## 4 Discussion

T cell exhaustion and dysfunction after repetitive BsAb treatment is known, but linkage to infectious complications is not clear [7]. Although 1‐week PAV stimulation reduced CEFX‐specific IFN‐γ CD8+ T cell responses in vitro, the T cells were still able to kill myeloma cell lines. One reason for this could be that whilst IFN‐γ production requires recognition of peptide on MHC on the APCs together with coreceptors such as CD86 and ICAM, BsAbs activate T cells and force T and myeloma cells together leading to transfer of perforin and granzyme B at the synapse. Thus, we can envisage that partially suppressed T cells would be able to kill myeloma cells, and conversely, T cells killing tumor may not be able to remove pathogens. Other explanations such as apoptosis of pathogen‐specific T cells and expansion of tumor‐specific T cells are possible, but unlikely as the frequency of tumor‐specific T cells would be very low in T cells from healthy individuals [11]. The T cells expanded, and upregulated activation and inhibitory molecules in vitro, suggesting that PAV was active and not inhibited by sBCMA.

In a small cohort of patients, all responding to BCMA‐targeted BsAbs, the number of pathogen‐specific T cells dropped. T cells were still in blood as they responded to anti‐CD3 stimulation. At Day 90, however, both pathogen‐specific and polyclonal T cell activation were significantly reduced compared to baseline.

Susceptibility to infection in patients treated with BCMA‐targeting BsAbs has been attributed to deletion of B cells [2], but our exploratory observations, although limited, could imply that T cell immunity to common pathogens is also affected. Whether this contributes to the increased frequency and severity of infections requires further investigations of possible mechanisms and more patients.

## 5 Conclusion

T cells cultured with a BCMA‐BiTEs and HMCLs killed myeloma cells, but showed reduced numbers of pathogen‐specific IFNγ+ CD8+ T cells. Pathogen‐specific memory T cells declined in the blood of four patients responding to BCMA‐targeted BsAb therapy. This initial observation should be followed up in a larger cohort of patients to investigate if and how reduction of T cell memory could be related to increased incidence of infections.

## Author Contributions

**Ida Steiro**: data curation, formal analysis, investigation, visualization, conceptualization, funding acquisition, writing – original draft, writing – review and editing. **Synne Stokke Tryggestad**: data curation, formal analysis, investigation, visualization, conceptualization, writing – review and editing. **Eli Svorkdal Hess**: data curation, investigation, writing – review and editing. **Hanne Hella**: data curation, writing – review and editing. **Pegah Abdollahi**: writing – review and editing. **Magne Børset**: supervision, writing – original draft, writing – review and editing. **Kari Lenita Falck Moore**: writing – review and editing. **Margrete Friestad**: data curation writing – review and editing. **Tobias S. Slørdahl**: conceptualization, supervision, funding acquisition, writing – original draft, writing – review and editing. **Anne Marit Sponaas**: conceptualization, supervision, project administration, writing – original draft, writing – review and editing.

## Funding

The study was supported by The Liaison Committee between the Central Norway Regional Health Authority (RHA) and the Norwegian University of Science and Technology (NTNU), The Joint Research Committee between St. Olavs Hospital and the Faculty of Medicine and Health Sciences, NTNU (FFU), St. Olav's Hospital, The Cancer Fund (Kreftfondet) at St. Olav's Hospital.

## Ethics Statement

The use of buffy coat from blood donors was approved by the Regional Ethics Committee (REK 2009/2245). Collection of patient samples was approved by the Regional Ethics Committee (REK 2023/562858). All donors gave informed consent.

## Conflicts of Interest

Ida Steiro has received honoraria for lecture; Pfizer. Tobias S. Slørdahl has received honoraria for lectures and educational material: Takeda, Celgene, Amgen, Johnsen & Johnsen/Janssen‐Cilag, Abbvie, Pfizer; Consultancy: Bristol Myers Squibb, GSK, Sanofi, Pfizer, Menarini Group; Advisory board consultancy: Amgen, Celgene, GSK, Johnsen & Johnsen/Janssen‐Cilag, Sanofi, Bristol Myers Squibb. The authors declare no competing interests.

## Supporting information




**Supporting Information file 1**: jha270388‐sup‐0001‐SuppMat.pdf

## Data Availability

The data may be available from the corresponding author upon request.

## References

[1] F. Mazahreh , L. Mazahreh , C. Schinke , et al., “Risk of Infections Associated With the Use of Bispecific Antibodies in Multiple Myeloma: A Pooled Analysis,” Blood Advances 7, no. 13 (2023): 3069–3074, 10.1182/bloodadvances.2022009435. PMC10331406 36857755

[2] K. A. Frerichs , C. P. M. Verkleij , M. V. Mateos , et al., “Teclistamab Impairs Humoral Immunity in Patients With Heavily Pretreated Myeloma: Importance of Immunoglobulin Supplementation,” Blood Advances 8, no. 1 (2023): 194–206, 10.1182/bloodadvances.2023011658. PMC10787247 38052042

[3] R. L. Goldstein , A. Goyos , C. M. Li , et al., “AMG 701 Induces Cytotoxicity of Multiple Myeloma Cells and Depletes Plasma Cells in Cynomolgus Monkeys,” Blood Advances 4, no. 17 (2020): 4180–4194, 10.1182/bloodadvances.2020002565. PMC7479952 32886754

[4] M. T. Esser , R. D. Marchese , L. S. Kierstead , et al., “Memory T Cells and Vaccines,” Vaccine 21, no. 5–6 (2003): 419–430, 10.1016/S0264-410X(02)00407-3. 12531640

[5] S. A. Calarota and F. Baldanti , “Enumeration and Characterization of Human Memory T Cells by Enzyme‐Linked Immunospot Assays,” Clinical & Developmental Immunology 2013 (2013): 637649, 10.1155/2013/637649. PMC3844203 24319467

[6] Y. Jiang , Y. Li , and B. Zhu , “T‐Cell Exhaustion in the Tumor Microenvironment,” Cell Death & Disease 6, no. 6 (2015): e1792–e1792, 10.1038/cddis.2015.162. PMC4669840 26086965

[7] N. Philipp , M. Kazerani , A. Nicholls , et al., “T‐Cell Exhaustion Induced by Continuous Bispecific Molecule Exposure Is Ameliorated by Treatment‐Free Intervals,” Blood 140, no. 10 (2022): 1104–1118, 10.1182/blood.2022015956. PMC10652962 35878001

[8] M. J. Friedrich , P. Neri , N. Kehl , et al., “The Pre‐Existing T Cell Landscape Determines the Response to Bispecific T Cell Engagers in Multiple Myeloma Patients,” Cancer Cell 41, no. 4 (2023): 711–725.e6, 10.1016/j.ccell.2023.02.008. 36898378

[9] H. Lee , M. Durante , S. Ahn , et al., “The Impact of Soluble BCMA and BCMA Gain on Anti‐BCMA Immunotherapies in Multiple Myeloma,” Blood 142, no. S1 (2023): 4688, 10.1182/blood-2023-188080.

[10] D. Vishwamitra , S. Skerget , D. Cortes , et al., “Longitudinal Correlative Profiles of Responders, Nonresponders, and Those With Relapse on Treatment With Teclistamab in the Phase 1/2 MajesTEC‐1 Study of Patients With Relapsed/Refractory Multiple Myeloma,” Blood 142, no. S1 (2023): 455–455, 10.1182/blood-2023-173550.

[11] O. Goodyear , K. Piper , N. Khan , et al., “CD8+T Cells Specific for Cancer Germline Gene Antigens Are Found in Many Patients With Multiple Myeloma, and Their Frequency Correlates With Disease Burden,” Blood 106, no. 13 (2005): 4217–4224, 10.1182/blood-2005-02-0563. 16144804

