# Supplementary material for: Reduced Pathogen‐Specific T Cell Response With BCMA‐Targeted T Cell‐Engaging Therapy to Multiple Myeloma
Source: EJHaem. 2026 Sep 21;7(5):e70388. doi: 10.1002/jha2.70388 (PMC13594405; doi:10.1002/jha2.70388)
Supplement: Supplementary file 1 [file JHA2-7-e70388-s001.pdf]

Supplemental Methods

Culture media and reagents. R10 medium: RPMI-1640 (Sigma-Aldrich, Saint-Louis, MO, USA) with 2 mM l-glutamine (Sigma-Aldrich), 20 µg/mL gentamicin (#453130, Sanofi, Paris, France) and 10% heat-inactivated fetal calf serum (FCS) (Gibco, Thermo Fisher Scientific, Waltham, MA, USA). R4 medium: X-VIVO 15 (BE02-060F) (Lonza, Allendale, NJ, USA) with 2 mM l-glutamine, 20 µg/mL gentamicin and 2% CTS Immune Cell Serum Replacement (A2596101) (Gibco). R2 medium: X-VIVO 15 with 2 mM l-glutamine and 20 µg/mL gentamicin. Pavurutamab (#155-21-947) and BiTE isotype control (#155-31-397) were obtained from ProSci (Poway, CA, USA). The BiTE isotype control includes an active CD3 binding site, whilst the target antigen of the isotype control is hen egg lysosome. Anti-human CD138 (#130-051-301) and anti-human CD3 microbeads (#130-050-101), and anti-human pan T cell isolation kit (#130-096-535), all from Miltenyi Biotec (Gaithersburg, MD, USA), were used to isolate cells from HMCL-PBMC cocultures. Anti-human interferon gamma (IFN-γ) ELISpot (#3420-2APT-10) was obtained from Mabtech (Nacka Strand, Sweden). CEFX peptide pool (#PM-CEFX1) containing peptides against *Clostridium tetani*, *Coxsackievirus B4*, *Haemophilus influenza*, *Helicobacter pylori*, *Human adenovirus 5*, *Human herpesvirus 1*, *Human herpesvirus 2*, *Human herpesvirus 3*, *Human herpesvirus 4*, *Human herpesvirus 5*, *Human herpesvirus 6*, *Human papillomavirus*, *Influenza A*, *JC polyomavirus*, *Measles virus*, *Rubella virus*, *Toxoplasma gondii*, and *Vaccinia virus*, was obtained from JPT Peptide Technologies (Berlin, Germany). Interleukin 2 (IL-2) (#200-02) and IL-7 (#200-07) were purchased from PeproTech (London, UK). The following conjugated antibodies against human antigens were purchased from BioLegend (San Diego, CA, USA): CD269-APC (BCMA, #357506), CD3-AF700 (#300424), CD4-FITC (#300506), CD8-APC/Cy7 (#344714), ICOS-PE (#313508), CD38-PE/Cy5 (#303508), TIGIT-BV605 (#372712) and PD-1-PE/Cy7

(#329918). Viability dye eFluor450 (#65-0863-14) was obtained from eBioscience (San Diego, CA, USA). Fixation buffer (FC004) was from R&D Systems (Abingdon, UK). The Western blot antibody against BCMA (#88183) was obtained from Cell Signaling Technology (Beverly, MA, USA) and the antibody against GAPDH (ab8245) was purchased from Abcam (Cambridge, UK). Human BCMA ELISA was from R&D (DY193).

Cell lines and culture conditions. The human myeloma cell lines JJN-3 (a gift from Dr. I.M. Franklin, University of Birmingham, Birmingham, UK), RPMI-8226 and U266 (both from ATCC, Rockville, MD, USA) were used for *in vitro* experiments. Stock cultures of the MM cell lines were cultured in RPMI-1640 supplemented with 2 mM l-glutamine. JJN-3 was supplemented with 10% FCS, RPMI-8226 with 20% FCS and U266 with 15% FCS. Cell culture media were replenished twice weekly. Cells were cultured at 37°C in a humidified atmosphere with 5% CO_2. New stock batches of the cell lines used were thawed at least every four months and were regularly tested for mycoplasma.

PBMCs. PBMCs from MM patients (BioBank1, St. Olav's Hospital, Trondheim, Norway) were isolated from blood samples and PBMCs from healthy blood donors (Department of Immunology and Transfusion Medicine, St. Olav's Hospital, Trondheim, Norway) were isolated from fresh buffy coats, both by density gradient centrifugation with Lymphoprep (Axis-Shield, Oslo, Norway), frozen with DMSO and stored in liquid N_2. Prior to use in experiments, patient PBMCs and healthy blood donor PBMCs were thawed and counted, and all samples were found to be >80% viable.

FACS. Cells were stained with viability dye and surface markers as indicated. Flow cytometry was performed using LSR II (BD Biosciences, Franklin Lakes, NJ, USA) with FACS Diva

software (BD Biosciences). Samples were analyzed with FlowJo V10.X (BD Biosciences). Gates were set on live cells with forward and side scatter. Complete gating strategies are found in Figure S7.

In vitro cell culture and cell isolation. PBMCs from healthy donors were seeded with JJN-3, RPMI-8226 or U266 myeloma cell lines and 13,75 ng/mL of either PAV or BiTE isotype control in an effector-to-target (MM cells) (E:T) ratio of 10:1 in R10 medium. Increasing concentrations of PAV was tested and the highest concentration examined, 13.75 ng/mL, was found to give the best response in terms of T cell expansion and activation. Fresh R10 media were replenished as needed during incubation (added to experimental and control wells to ensure comparable conditions). The coculture was incubated for a total of seven days at 37°C with 5% CO_2. No myeloma cells were added throughout cultivation. Following incubation, the experimental setup diverged for the different assays. For *in vitro* T cell ELISpot and cytotoxicity assay (restimulation setup), T cells were isolated from the coculture by two-step magnetic separation. First, CD138-positive cells were removed by positive selection, followed by isolation of T cells by negative selection. For the baseline cytotoxicity assay, T cells were isolated directly from thawed PBMCs prior to cultivation. Purity of T cells was >80%.
The T cells targeted by PAV and myeloma cells expanded during the culture period and more T cells were harvested from the cultures stimulated by PAV than isotype control at day seven (Figure S2).

Isolation of antigen-presenting cells (APCs). For the *in vitro* T cell ELISpot assay, APCs were isolated from frozen PBMCs from the same donor as used for coculture of PBMCs and MM cells. T cells were removed by positive selection with CD3 beads. The purity of APCs

(depletion of T cells) was >80%. All isolation procedures were performed according to the manufacturer´s protocol.

Cytotoxicity assay. HMCLs were labelled with carboxyfluorescein succinimidyl ester (CFSE) dye (10 nM) using the CellTrace™ CFSE Cell Proliferation Kit (C34554) (Invitrogen, Thermo Fisher Scientific) for 20 minutes at room temperature. Hanks' balanced salt solution (Sigma-Aldrich) with 2% FCS was added to quench unbound dye. Cells were washed, resuspended in R10 medium and seeded with isolated T cells as described at indicated E (T cells):T (MM cells) ratios (10:1, 5:1 and 1:1) in the presence of PAV or control antibody. Following 18-24-hour incubation at 37°C with 5% CO_2, cells were stained with viability dye eFluor 450 and anti-CD3 antibody prior to analysis. CFSE-positive cells were identified as myeloma cells during gating. Gating strategy is shown in Figure S5.

Patient samples. Patients with MM eligible for treatment with BsAbs at three academic hospitals in Norway were asked to participate in the study. All patients were BsAb-naïve. Peripheral blood samples were collected at baseline, i.e., before start of therapy, and after 30 and 90 days (+/- 7 days) on treatment. The standard treatment schedule with elranatamab or teclistamab included an initial step-up cycle, and subsequently, doses were given every other week. Peripheral blood samples were collected at baseline, i.e., before start of BsAb-treatment, and after approximately 30 and 90 days on treatment. Patient information is shown in Table 1. Relevant clinical data from the patient records were collected, including data from the national immunization registry. The follow-up period was defined from start of treatment with bispecific antibody until 1st of June 2024 or start of new treatment, whatever occurred first.

*T cell memory assay in patients/T cell ELISpot***.** PBMCs were thawed and seeded approximately at $2*10^6$ cells/mL in R4 medium with CEFX 3 µg/mL. Cytokines IL-2 20 U/mL and IL-7 5 ng/mL were added after two and five days of incubation. Cells were cultivated for a total of ten days at 37°C with 5% CO_2 before IFN-γ-producing T cells were quantified using the ELISpot assay, according to the manufacturer's protocol. Briefly, $5*10^4$ cells/well were seeded in a 96-well ELISpot plate pre-coated with anti-IFN-γ antibody in R2 medium. Cells were stimulated with 3 µg/mL CEFX peptide pool, 0,3 µg/mL anti-CD3 mAb or media only. Plates were incubated at 37°C with 5% CO_2 for 21-24 hours followed by ALP-linked immunosorbent detection. The AID vSpot Spectrum was used for counting spots. Results are expressed as number of spots/$5*10^4$ seeded cells. Fresh R10 media were replenished when needed during the expansion and differentiation period.

M-protein analysis. Quantification of monoclonal protein (M-protein) was based on total protein levels and was performed by using the tangent skimming method (1).

Immunoblotting. Cells were harvested and lysed without any prior stimulation. Immunoblotting method was performed as previously described (2). Images were acquired using Odyssey Fc imager (LI-COR Biosciences, Lincoln, Nebraska, USA) and analyzed with Image Studio Software (LI-COR Biosciences).

Soluble BCMA detection. $2x10^6$ PBMCs were incubated with 13,75 ng/ml of either PAV or control antibody in an E:T ratio of 10:1 in R10 medium. Supernatants were harvested and centrifuged at 5000 RPM at baseline (day zero), and after one and three days. Soluble BCMA was determined by ELISA following the manufacturer`s instructions.

Statistical analysis. Statistical analyses were performed using GraphPad Prism 10.0 (GraphPad Software). $p < 0.05$ was considered statistically significant. Unpaired Student´s was used for comparing two groups and one-way ANOVA with Tukey´s multiple comparisons test was used for analyzing three groups.

References

1. Schild C, Wermuth B, Trapp-Chiappini D, Egger F, Nuoffer JM. Reliability of M protein quantification: comparison of two peak integration methods on Capillarys 2. Clin Chem Lab Med. 2008;46(6):876–7.
2. Slørdahl TS, Denayer T, Moen SH, Standal T, Børset M, Ververken C, et al. Anti-c-MET Nanobody - a new potential drug in multiple myeloma treatment. Eur J Haematol. 2013 Nov;91(5):399–410.

Supplemental Figures and Tables

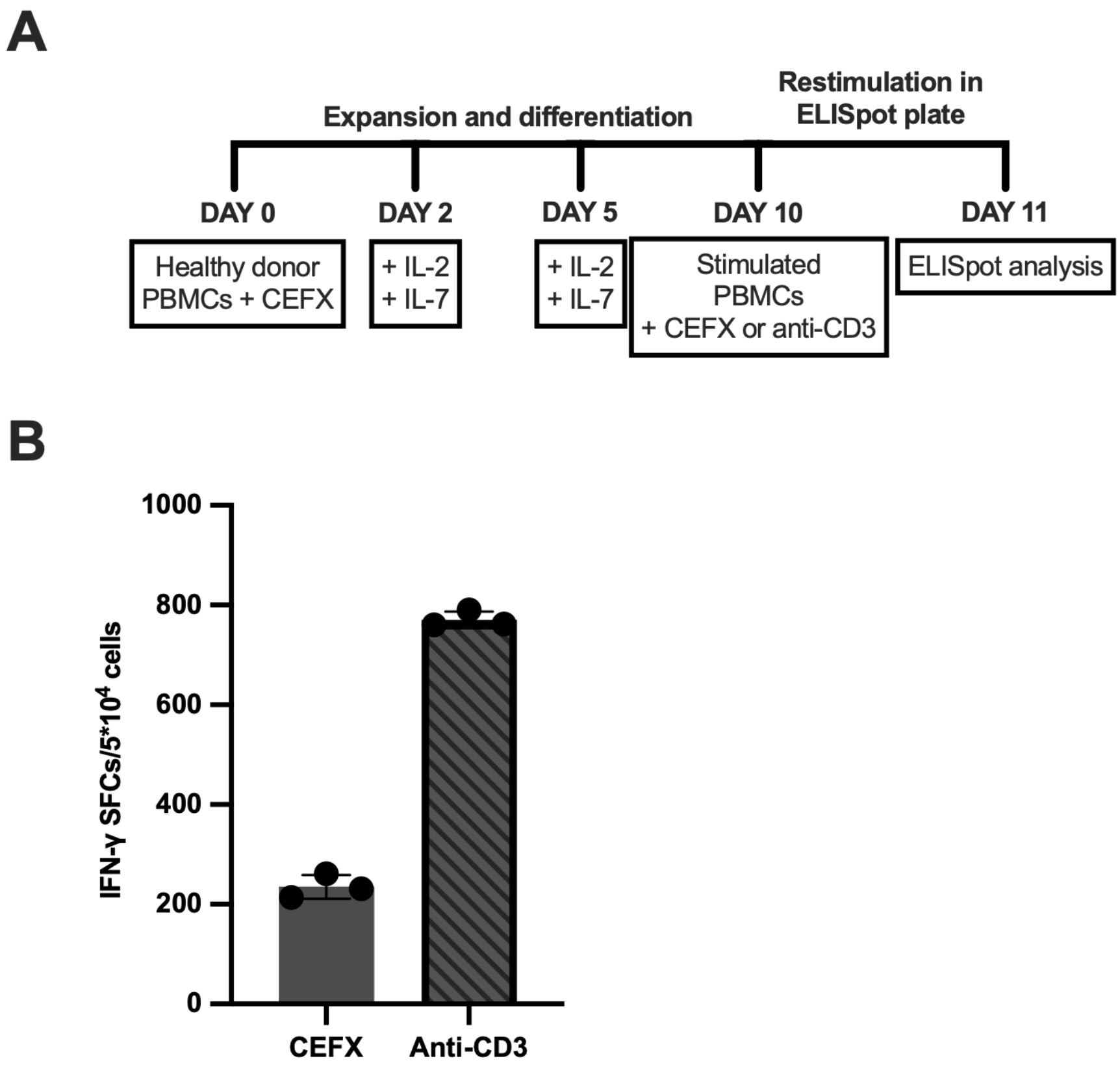

Supplemental Figure 1. PBMC positive control in the T cell ELISpot assay.
(A) Experimental setup for (B). PBMCs isolated from healthy donors were stimulated with CEFX peptide mix on day 0. IL-2 and IL-7 were added twice during cultivation. After 10 days, the cells were harvested and seeded in ELISpot plates following restimulation with CEFX or anti-CD3. After 24 hours incubation, the plate was developed and spots counted on AID vSpot Spectrum. **(B)** Memory T-cells in healthy donors. The figure shows mean + SD of three technical replicates from one of at least three independent experiments. SFC: Spot forming colony.

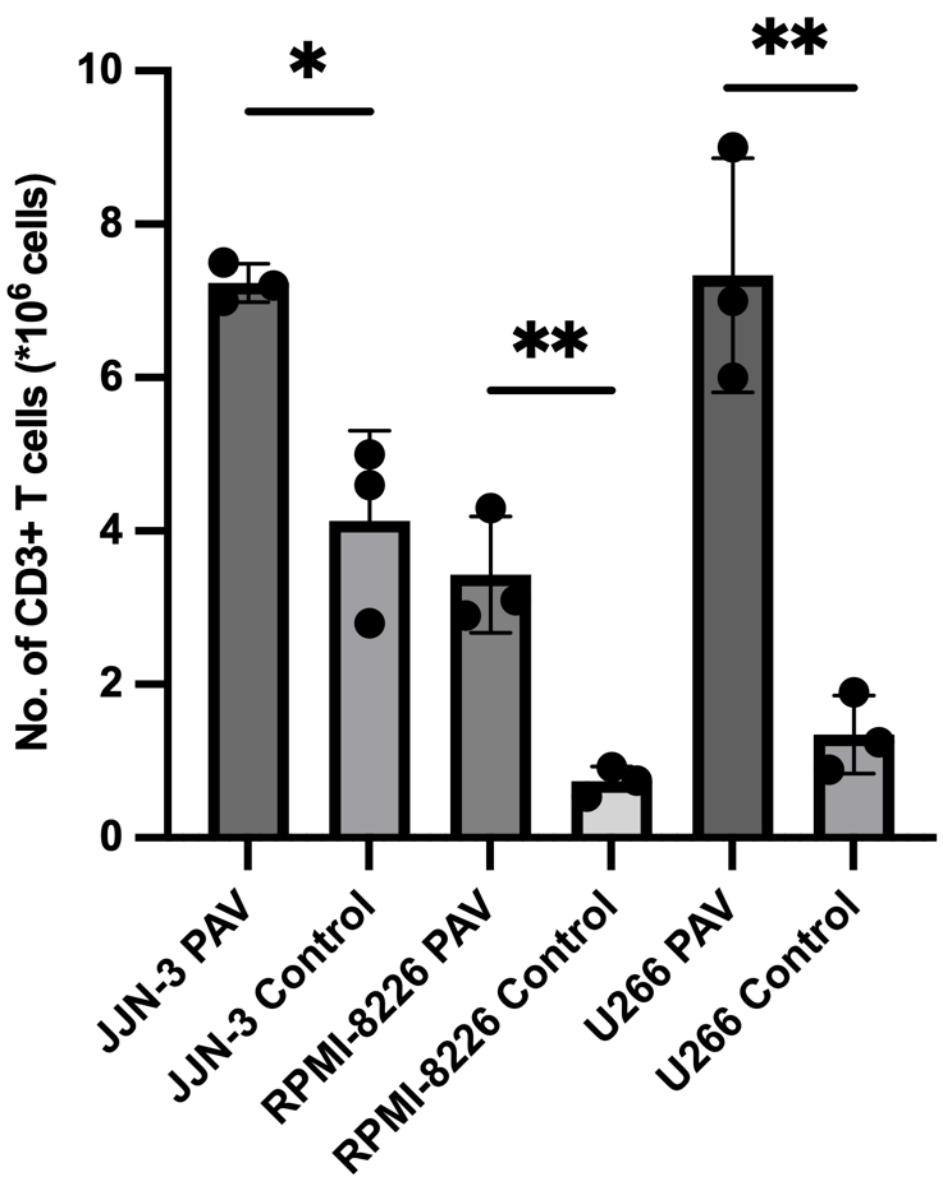

Supplemental Figure 2. Expansion of T cells after stimulation with PAV. PBMCs were stimulated with pavurutamab (PAV) or BiTE isotype control antibody (control) and HMCL as described in Figure 2. T cells were isolated as described and stained with anti-CD3 and analysed on LSRII. Figure shows numbers of CD3+ T cells havested from three independent experiments with T cells from the same donor.

A

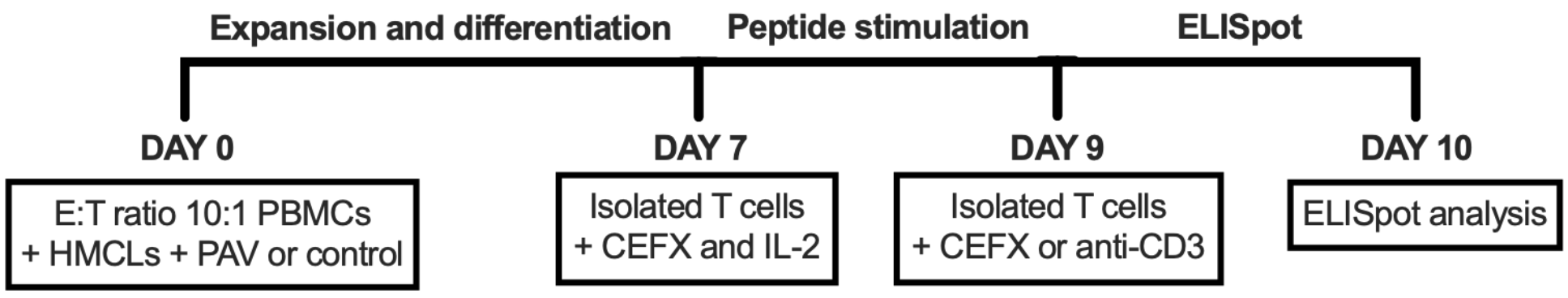

B

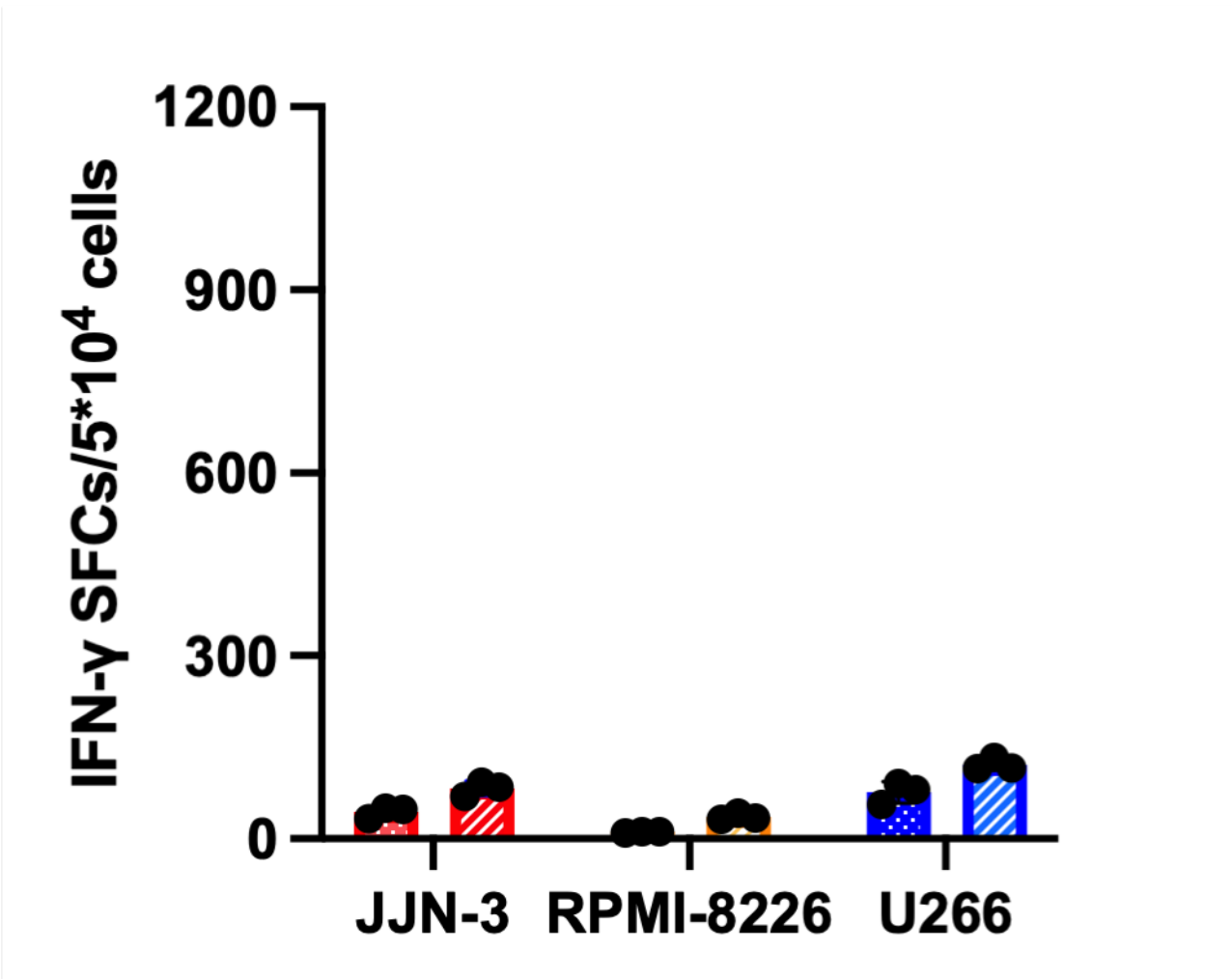

Supplemental Figure 3. Minor memory T cell response in the absence of syngeneic APCs following PAV stimulation. (A) Experimental setup for (B). PBMCs were seeded with HMCLs and pavurutamab (PAV) on day 0. After 7 days incubation, T cells were isolated and seeded with CEFX peptide pool and IL-2. After 2 days incubation, cells were harvested and restimulated with CEFX or CD3 in ELISpot plates. Following overnight incubation, plate was developed and spots counted on AID vSpot Spectrum. **(B)** T cells isolated from coculture with JJN-3, RPMI-8226 and U266 myeloma cell lines, respectively. Bar graphs shows PAV-stimulated T cells restimulated with CEFX (dots) or CD3 (stripes). SFC: Spot forming colony.

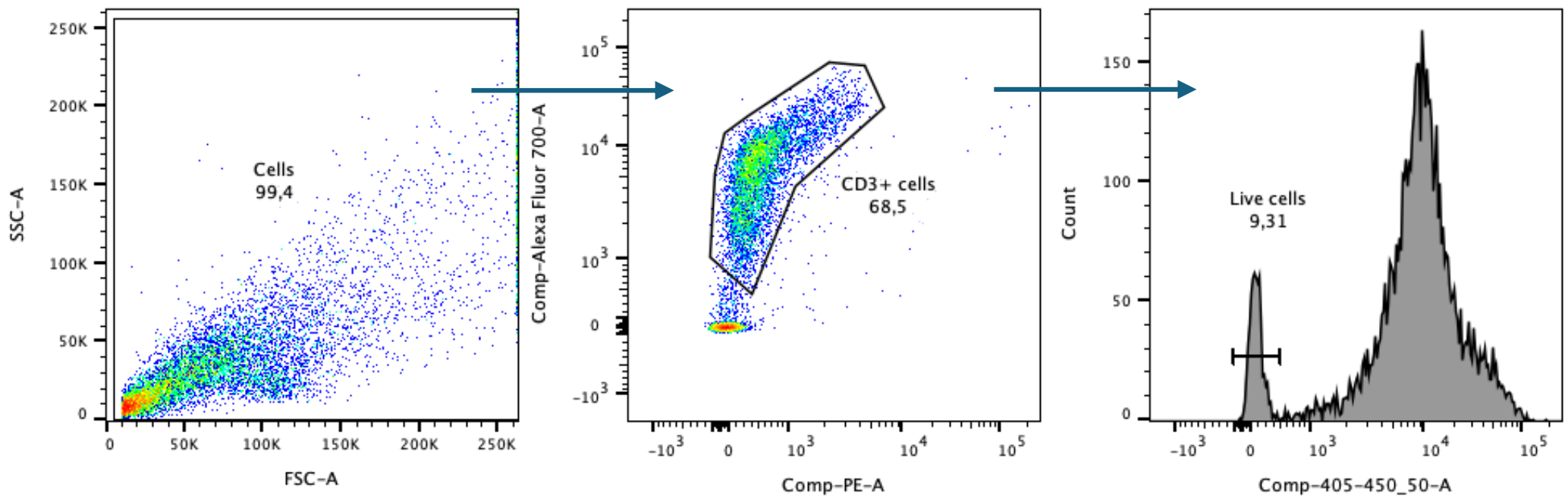

Supplemental Figure 4. T cell viability after second PAV stimulation with the JJN-3 cell line. PBMCs were seeded with JJN-3 and pavurutamab (PAV) on day zero. After seven days of incubation, T cells were isolated from culture and seeded with PAV and fresh JJN-3 cells from stock culture. After another seven days of incubation, T cells were again isolated and analyzed by flow cytometry. The figure shows from left to right gating of all cells, gating of CD3+ cells (CD3+CD138- (AF700+PE-)), and percentage live cells of the gated CD3+ cells (eFluor450+ (405-450/50)).

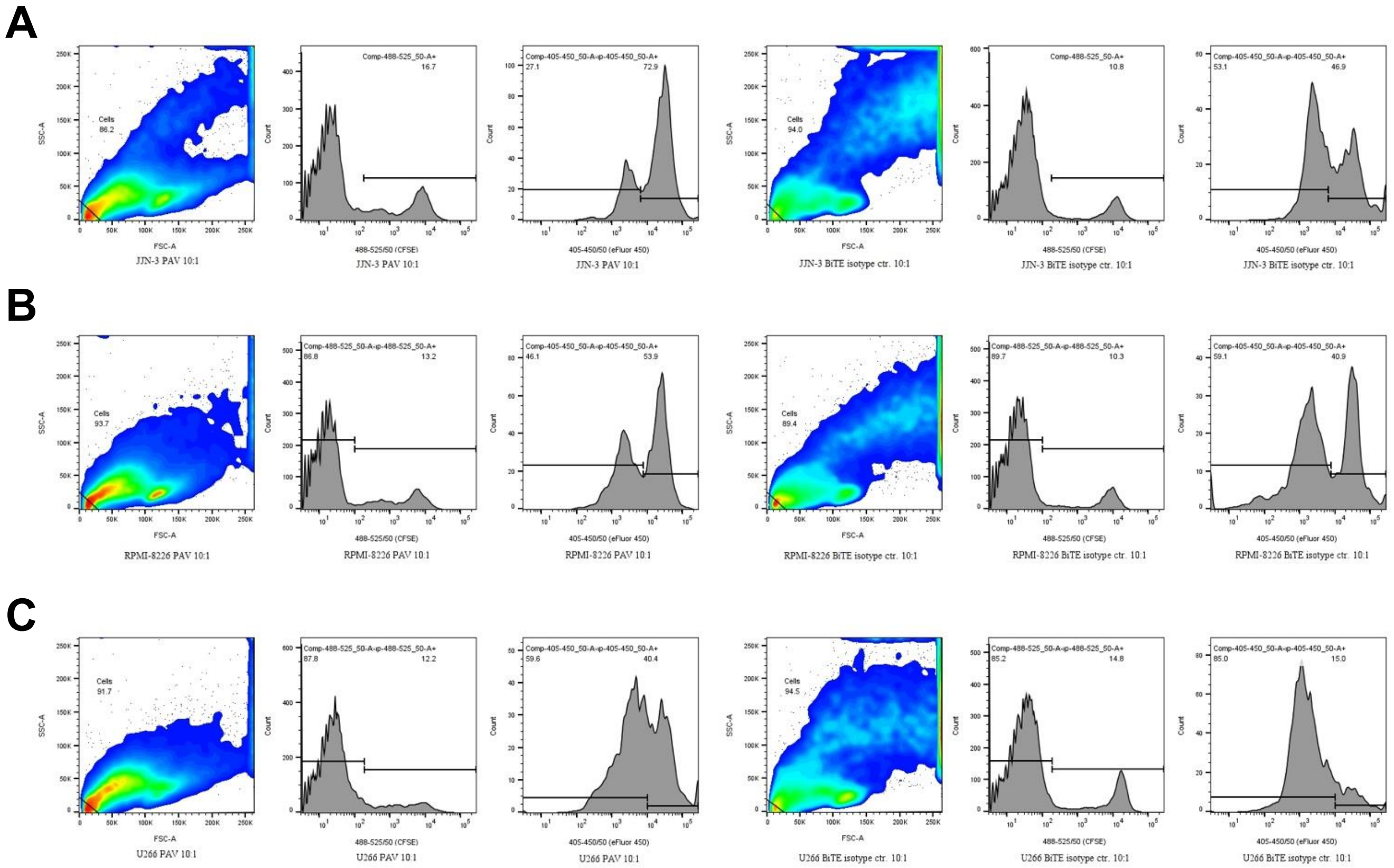

Supplemental Figure 5. Gating strategy cytotoxicity assay. Flow cytometry representative plots displaying gating strategy used to quantify dead myeloma cells in 10:1 E:T ratio cocultures of isolated T cells and JJN-3 **(A)**, RPMI-8226 **(B)** and U266 **(C)** myeloma cells, respectively, restimulated with pavurutamab (left) and BiTE isotype ctr. (right). The same strategy was used on all E:T ratios, and in both baseline and restimulation experiments.

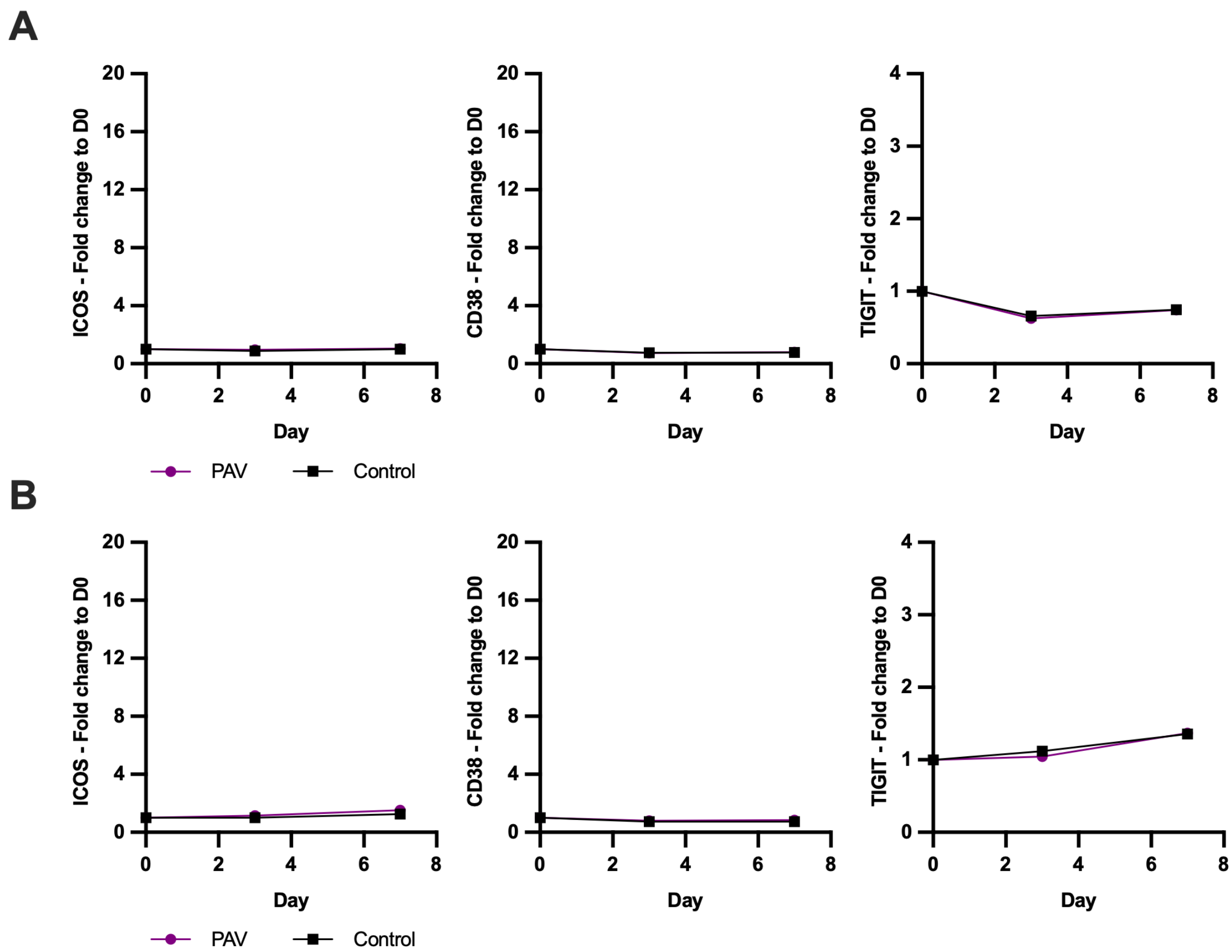

Supplemental Figure 6. PAV does not stimulate PBMCs in the absence of HMCLs. PBMCs from healthy donors were stimulated with pavurutamab (PAV) or BiTE isotype control antibody (control). Cells were harvested and stained at baseline (day 0), and after 3 and 7 days cultivation for quantitative flow cytometry analysis to evaluate subsets of CD8+ **(A)** and CD4+ **(B)** T cells as indicated. ICOS, CD38 and TIGIT surface markers were investigated. Data are shown as MFI fold change to baseline samples. D0: Day 0.

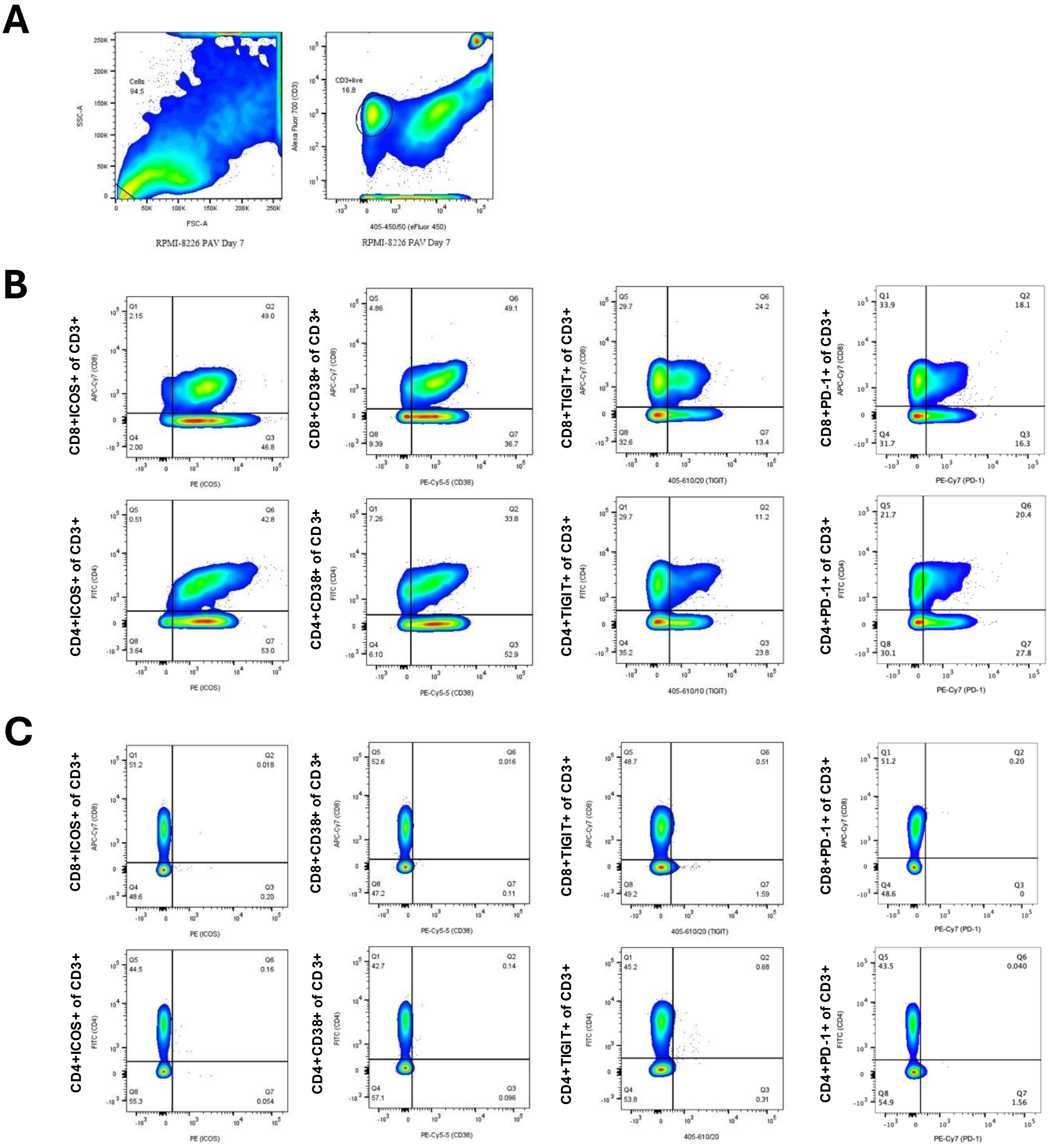

Supplemental Figure 7. Gating strategy 1-week PAV stimulation. Flow cytometry representative plots indicating gating strategy used to gate CD3+ live cells **(A)**, and CD4+ and CD8+ subpopulations of CD3+ cells **(B)**. Corresponding FMO controls are shown in **(C)**. The displayed sample is PBMC-RPMI-8226 coculture stimulated with PAV and harvested on day 7. The same gating strategy was applied to all cocultures, including all HMCLs used and regardless of PAV or BiTE isotype ctr. stimulation. FMO: Fluorescence minus one.

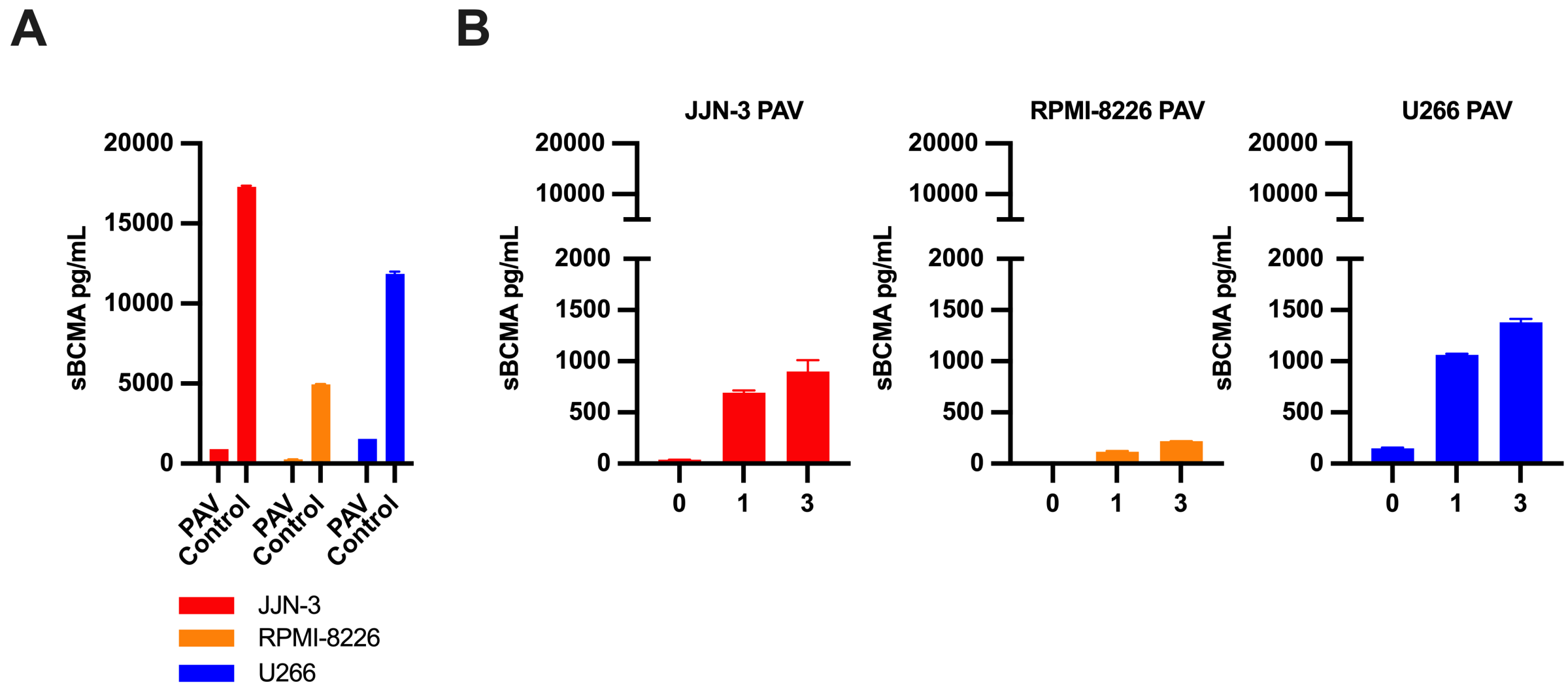

Supplemental Figure 8. Soluble BCMA in cocultures. PBMCs and cell lines JJN-3, RPMI-8226 and U266, respectively, were cultured with pavurutamab (PAV) or BiTE isotype control antibody (control). Supernatants were harvested at baseline (day 0), and after one and three days cultivation. sBCMA was determined by ELISA. **(A)** sBCMA in cocultures with PAV or control harvested after three days cultivation. **(B)** sBCMA in cocultures with PAV analyzed at baseline, and after one and three days cultivation. sBCMA: Soluble BCMA.

Supplemental Table 1. Overview of percentage dead HMCLs in experiments from the same donor as shown in Figure 3.

		Baseline						Restimulation					
		Experiment 1		Experiment 2		Experiment 3		Experiment 1		Experiment 2		Experiment 3	
	E:T ratio (T cells :HMCL)	PAV	Control	PAV	Control	PAV	Control	PAV	Control	PAV	Control	PAV	Control
**JJN-3**	**10:1**	**85.5**	14.6	**61,7**	11,3	**61,9**	11	**72.9**	46.9	**61.2**	39.5	**60.4**	48.9
	**5:1**	**86.4**	10.3	**60,8**	9,26	**63,4**	8,07	**78.1**	48.7	**59.1**	33.1	**59.3**	42.2
	**1:1**	**65.4**	6.79	**46,3**	6,8	**44,4**	7,65	**70.1**	40.0	**60.6**	31.0	**66.3**	40.1
**RPMI-8226**	**10:1**	**68.3**	35.2	**49,4**	29,9	**40,8**	29,6	**53.9**	40.9	**50.9**	52.9	**54.9**	52.1
	**5:1**	**67.3**	32.0	**46,6**	28,6	**49,1**	30,7	**49.0**	38.4	**47.8**	42.7	**52.4**	47.0
	**1:1**	**41.8**	30.6	**34,4**	26,5	**38,1**	27,4	**51.0**	37.6	**48.4**	43.4	**50.1**	47.9
**U266**	**10:1**	**41.3**	10.0	**15,5**	12	N/A	N/A	**40.4**	15.0	**22.0**	11.0	**27.8**	9.55
	**5:1**	**14.1**	11.2	**18,8**	5,4	**19,9**	4,99	**29.7**	11.7	**27.4**	11.9	**19.7**	9.50
	**1:1**	**28.4**	11.9	**8,09**	4,72	**9,31**	4,24	**15.1**	10.6	**14.1**	12.2	**10.4**	9.09
